# Highly potent VEGF-A-antagonistic DARPins as anti-angiogenic agents for topical and intravitreal applications

**DOI:** 10.1007/s10456-012-9302-0

**Published:** 2012-09-15

**Authors:** Andreas Stahl, Michael T. Stumpp, Anja Schlegel, Savira Ekawardhani, Christina Lehrling, Gottfried Martin, Maya Gulotti-Georgieva, Denis Villemagne, Patrik Forrer, Hansjürgen T. Agostini, H. Kaspar Binz

**Affiliations:** 1 Universitäts-Augenklinik Freiburg, Killianstrasse 5, 79106 Freiburg, Germany; 2 Molecular Partners AG, Wagistrasse 14, 8952 Zürich-Schlieren, Switzerland

**Keywords:** Binding protein, DARPin, Ophthalmology, Repeat protein, VEGF

## Abstract

The next-generation ophthalmic anti-VEGF therapeutics must aim at being superior to the currently available agents with regard to potency and improved drug delivery, while still being stable and safe to use at elevated concentrations. We show here the generation of a set of highly potent VEGF-A antagonistic DARPins (designed ankyrin repeat proteins) delivering these properties. DARPins with single-digit picomolar affinity to human VEGF-A were generated using ribosome display selections. Specific and potent human VEGF-A binding was confirmed by ELISA and endothelial cell sprouting assays. Cross-reactivity with VEGF-A of several species was confirmed by ELISA. Intravitreally injected DARPin penetrated into the retina and reduced fluorescein extravasation in a rabbit model of vascular leakage. In addition, topical DARPin application was found to diminish corneal neovascularization in a rabbit suture model, and to suppress laser-induced neovascularization in a rat model. Even at elevated doses, DARPins were safe to use. The fact that several DARPins are highly active in various assays illustrates the favorable class behavior of the selected binders. Anti-VEGF-A DARPins thus represent a novel class of highly potent and specific drug candidates for the treatment of neovascular eye diseases in both the posterior and the anterior eye chamber.

**Electronic supplementary material:**

The online version of this article (doi:10.1007/s10456-012-9302-0) contains supplementary material, which is available to authorized users.

## Introduction

The vascular endothelial growth factor (VEGF) cytokine family members are key controllers of angiogenesis and lymph angiogenesis [1]. VEGF-A is a major regulator of angiogenesis, signaling, amongst others, via VEGF receptor 2, a type III receptor tyrosine kinase [2]. Blocking VEGF-A and especially its predominant splice variants VEGF-A165 and VEGF-A121 leads to nearly complete inhibition of blood vessel growth [3]. Both in tumor therapy, where new blood vessels are required to support tumor growth, and in ophthalmology, where uncontrolled blood vessel growth and vascular leakage leads to loss of vision, VEGF-blocking drugs have shown significant clinical benefits. Consequently, there are a number of anti-VEGF drugs approved for clinical application [4, 5] or in clinical testing [6, 7]. In ophthalmology, currently two VEGF-antagonistic drugs are approved for the treatment of wet age-related macular degeneration (wet AMD) worldwide: ranibizumab, a Fab antibody fragment binding the most relevant splice variants of VEGF-A [8, 9], and pegaptanib, a PEGylated aptamer that binds the heparin-binding domain of VEGF-A [10].

The main limitations of the current ophthalmic VEGF inhibitors are related to the need for frequent intravitreal injection. The requirement for monthly or bimonthly injection regimens with the currently available substances [11, 12] are due to a relatively low efficacy and fast ocular clearance of these drugs. As a consequence, an individual patient’s risk for injection-associated complications like endophthalmitis or retinal tears increases cumulatively over a prolonged treatment period and not only represents a burden for the affected patient but also leads to substantial healthcare costs associated with the repeated injections [13, 14].

New VEGF inhibitor development in ophthalmology should thus aim at providing highly potent molecules that can be delivered safely to patients with posterior and potentially also anterior segment eye disease. With regard to improved drug delivery and safety, drug delivery by eye-drops [15, 16] or increasing activity to achieve prolonged intraocular efficacy after intravitreal injection [6] appear to be the most promising approaches. In addition, new potential inhibitors should be robust to handle in order to facilitate their evaluation in different model systems and to facilitate high-concentration formulation development for clinical applications.

DARPins (designed ankyrin repeat proteins) are a new class of binding proteins that fulfill all the above criteria [17]. They are derived from the abundant protein family of naturally occurring ankyrin repeat proteins comprising human Ankyrin or human GA-binding protein [18]. Previous work from our group has generated libraries of DARPins of varying repeat numbers using protein engineering and recombinant DNA technology [18]. Individual members of these libraries were well-expressed in soluble form in the cytoplasm of *E. coli* and were found to exhibit very favorable thermal and thermodynamic stability [18–21]. In a fashion that is similar or superior to what is possible with antibodies, ribosome or phage display can be used to obtain specific high-affinity binding DARPins against desired target molecules [17, 22, 23]. Importantly, in addition to good specificity and strong affinity, the generated DARPins exhibit a very high robustness including high thermal and thermodynamic stability and high solubility, which allows for straight-forward high-concentration formulation development and the evaluation and use of new application routes. Due to the absence of additional effector functions, DARPins appear especially advantageous for the design of antagonistic anti-cytokine drugs [24]. In the present study, we evaluate DARPins as the next generation anti-VEGF-A drugs.

## Results and discussion

In order to produce VEGF-A inhibiting DARPins, a pool of putative VEGF-A binding DARPins was generated using ribosome-display selections from naïve DARPin libraries [17]. From this pool, individual DARPins were screened in a crude extract ELISA to identify potent VEGF-A binding DARPins. Binding DARPins were then expressed, purified and characterized by different ELISAs and cellular assays. Selected DARPins were further analyzed for penetration of ocular tissues upon intravitreal injection in a mouse model. For the study of efficacy in vivo, one DARPin was applied intravitreally to assess its potential to inhibit fluorescein extravasation in a rabbit model of retinal vascular leakage. The potential of topically applied DARPin to block laser-induced retinal neovascularization was assessed in a rat model. Similarly, the potential of topically applied DARPin to prevent the growth of blood vessels in eyes with a corneal suture was assessed in rabbits. Importantly, we used different DARPins for the individual experiments illustrating that a set of several powerful VEGF-A inhibiting DARPins has been generated.

### Rapid generation of a panel of anti-VEGF-A DARPin drug candidates

**Fig. 1:**
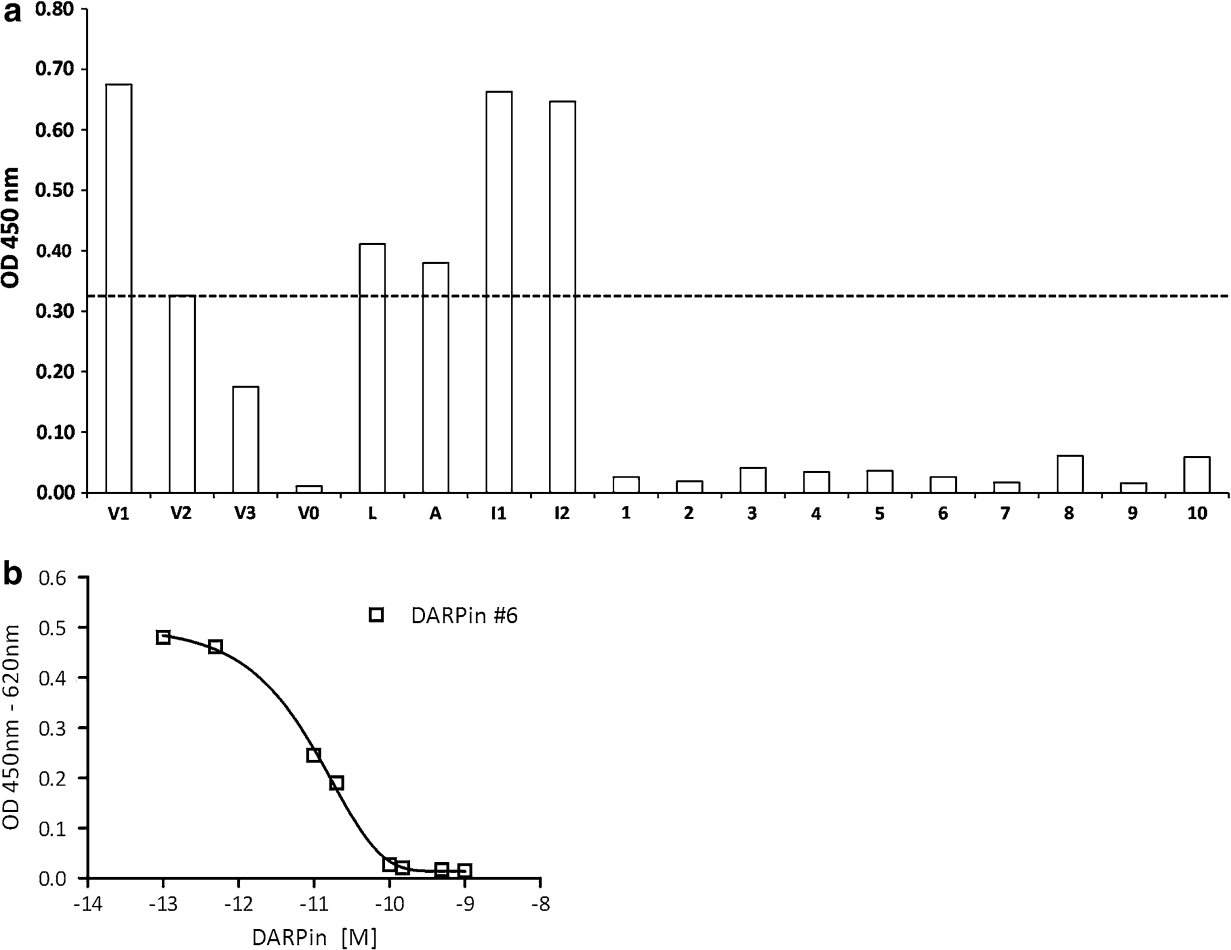
Screening for potent VEGF-A-binding DARPins and characterization of their affinities by sandwich inhibition ELISA (Quantikine; see “Materials and methods” section). **a** Screening for quantitative VEGF-A-inhibiting DARPins. Numbers 1–10 represent 10 different VEGF-A binding DARPins used in this screening assay. *L* represents Lucentis (ranibizumab), *A* represents Avastin (bevacizumab), I1 and I2 represent two non-binding DARPin isotype controls. V1, V2, V3, and V0 represent the signals obtained for 25, 12.5, 6.25, and 0 pM VEGF-A, respectively, applied without any inhibitor. The *dashed line* represents the signal obtained with free 12.5 pM VEGF-A, corresponding approximately to the equivalent of 50 % inhibition. **b** IC50 analysis of DARPin #6 in the identical assay as **a**. DARPin #6 is displayed as one VEGF-binding DARPin representative; *similar curves* were obtained for other anti-VEGF DARPins identified in **a**

Consistent with previous publications [17, 25, 26], target-binding DARPins from naïve DARPin libraries could rapidly be enriched using ribosome display. To guarantee high-affinity binding, three standard selection rounds were performed followed by three consecutive off-rate selection rounds followed by a collection round. Importantly, no additional randomization was applied during the selection process and a proof-reading DNA-polymerase was used for DNA amplifications. The resulting DNA pools were screened for VEGF-A binders by crude extract ELISA. Binders with strong ELISA signal were further characterized. The identified candidates were expressed in *E. coli* and purified from the soluble fraction using described methods (see “Materials and methods”). Expression levels were comparable to previously published DARPins and in the range of 200 mg expressed protein per liter shake-flask culture using LB-Lennox medium supplemented with 1 % glucose and using *E. coli* XL-1 Blue as expression strain. Strong interaction of the DARPins with VEGF-A165 was confirmed by ELISA. A competition assay showed that the DARPins interact well with both VEGF-A121, and VEGF-A165 (of human, dog, mouse, and rabbit), but not with VEGF-C. After purification, anti-VEGF-A DARPins were analyzed in more detail using a Quantikine sandwich ELISA (Fig. 1). In this assay, human VEGF-A is incubated with either a DARPin or controls and then applied to a plate which is pre-coated with a monoclonal anti-VEGF-A antibody. VEGF-A binding to the plate is then detected using a polyclonal anti-VEGF-A-HRP conjugate. Strong VEGF-A binders thus quantitatively reduce the ELISA signal compared to controls. Our results showed that all DARPins tested induced strong signal suppression of more than 50 % (Fig. 1), even when using only 25 pM (monomer) VEGF-A, whereas the isotype controls (i.e. non-binding DARPins) were not affecting the signals. This indicates that the affinity of these DARPins tested is at least K_D_ = 25 pM and that the chosen selection strategy led to a panel of highly potent anti-VEGF-A DARPins. As most DARPins showed inhibited signal down to background (Fig. 1), the potency of DARPin #4 was analyzed in more detail by performing the Quantikine experiment with varying DARPin concentrations (Fig. 1). An apparent IC50 value of 10 pM was derived by fitting the observed values. Note that in this experiment, the VEGF-A concentration (20 pM) is limiting exact affinity determination, as the DARPin titrates the amount of VEGF-A, indicating that the effective IC50 may be below 10 pM for DARPin #4. A more accurate determination of the DARPin affinity is limited by the detection limit of the Quantikine assay. The low picomolar anti-VEGF-A affinity of the selected DARPins was further confirmed by surface Plasmon resonance (SPR), where the detection system similarly works at its detection limits due to very slow off-rates. Also, we could show that by binding to VEGF-A, the DARPins block binding of VEGF-A to its receptor VEGFR-2, similar to Lucentis (see Supplementary Figure 1).

In summary, a large number of highly potent and specific VEGF-A binders could be isolated after seven rounds of ribosome display without the necessity of introducing novel randomizations for affinity maturation. To the best of our knowledge these are the highest affinity binders ever reported that were isolated by an in vitro selection process from naïve libraries without additional randomization. Picomolar affinity VEGF-A binders are thus readily available in the naïve DARPins library.

### Functional in vitro assays confirming highly potent VEGF-A antagonism

**Fig. 2:**
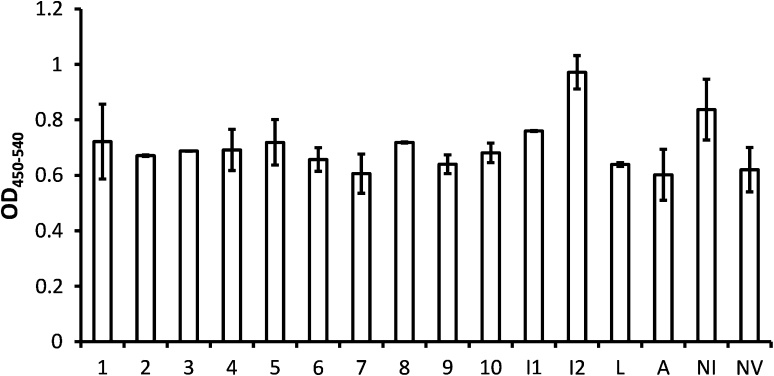
Cellular screening for DARPins inhibiting VEGF-induced phosphorylation of VEGFR-2. VEGF-A (1.4 nM) was pre-incubated with 1.4 nM of different purified DARPins (#1–#10), isotype (negative) control DARPins (I1 and I2), as well as the controls ranibizumab (Lucentis, *L*), bevacizumab (Avastin, *A*). The signal of PBS (*NI* non-inhibited) and the signal obtained without addition of VEGF-A (NV) were used as controls

**Fig. 3:**
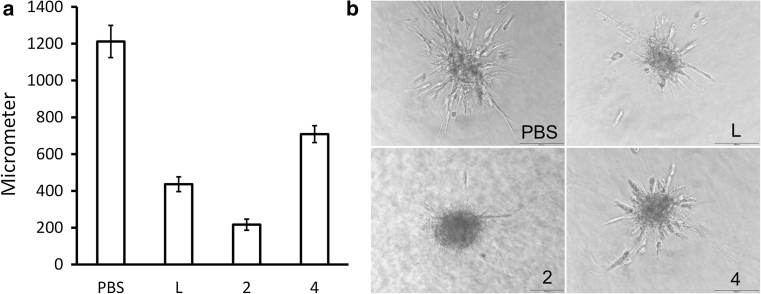
HUVEC spheroid sprouting inhibition with DARPins. HUVEC spheroids were incubated with VEGF-A, mixed with either PBS, or test substances. Test substances were DARPins #2 and #4, and ranibizumab (2, 4, and *L* (Lucentis), respectively). **a** Average accumulated total sprout length per spheroid. All groups (*L*, 2 and 4) are statistically different from PBS controls using ANOVA analysis and Tukey Multiple Comparison post hoc test (*p* < 0.001). **b** Representative pictures of sprouting spheroids. The isotype control DARPin I2 showed equivalent results as the PBS control

To confirm the potency of selected DARPins, we performed a VEGF-receptor-2 (VEGF-R2) phosphorylation-inhibition cell culture assay (Fig. 2) as well as a human umbilical vein endothelial cell (HUVEC) spheroid sprouting assay (Fig. 3). In the VEGF-R2 phosphorylation assay, the ten VEGF-binding DARPins as well as controls (Isotype DARPin I1 and I2 as negative controls [18]; bevacizumab and ranibizumab as positive controls) were used at a fixed concentration of 1.4 nM in combination with 1.4 nM VEGF-A. Maximal stimulation (PBS added to VEGF-A) and no stimulation (PBS, no VEGF-A used) were used to illustrate the assay window. The results demonstrated that all DARPins and the positive controls are able to suppress VEGF-A induced phosphorylation of VEGFR-2, whereas isotype controls do not affect its phosphorylation (Fig. 2). Overall, some of the selected DARPins showed inhibition potency in the range of ranibizumab and bevacizumab in this assay. These data were confirmed in a spheroidal model of in vitro sprouting angiogenesis [27–29]. In this assay, anti-VEGF-A DARPins potently suppressed endothelial cell sprout formation. Similar to what was observed in the phosphorylation assay, some of the DARPin candidates blocked sprouting very potently, similar to or better than ranibizumab, while the isotype control DARPins showed no effect. These cellular assays confirm that a large number of VEGF-A antagonists was generated, some of which exhibit very high potency in vitro. Note that for both cellular assays, VEGF-A has to be used at 8–70 times higher concentrations than the one used in the Quantikine assay to reliably induce VEGF receptor phosphorylation or spheroid sprouting. Consequently, potency values are limited by the concentration of human VEGF-A used and are thus underestimated.

### Ocular penetration and retention of intravitreally injected DARPins

The potential of DARPins to penetrate into different compartments of the eye after intravitreal injection was assessed. These experiments are important as intravitreal application represents the current standard approach to deliver anti-VEGF-A agents for the treatment of angio-proliferative retinal disease like wet AMD [8, 9]. Two Alexa-labeled DARPins, one binding VEGF and one isotype control DARPin, were injected intravitreally into mouse eyes and the distribution of fluorescently labeled DARPins was analyzed at various time points on whole eye cross-sections. Supplementary Figure 2 shows representative examples of DARPin distribution after intravitreal injection. Notably, fluorescently labeled DARPins penetrated into the retina and could be detected throughout all retinal layers. No difference was observed between the two DARPins tested. Images from control-injected eyes acquired with identical exposure time showed no fluorescent signal in the retina, thus ruling out auto-fluorescent artifacts. These results demonstrate that intravitreal injection of DARPins is a suitable way to target retinal disease.

### Vascular leakage inhibition by intravitreal DARPin application

Having shown that intravitreally injected DARPin can penetrate into the retina, the potential of anti-VEGF-A DARPins to treat retinal angiogenic disorders was investigated. VEGF-A is the most potent angiogenic mediator identified to date [30–32] and is crucially involved in mediating the proliferative phase of diabetic retinopathy and age-related macular degeneration. In addition, it induces vascular hyperpermeability leading, for example, to central macular edema in patients with diabetic retinopathy [33] or retinal vein occlusion [34]. We utilized the permeability-inducing properties of VEGF-A in a rabbit model of VEGF-A induced vascular leakage to investigate the effect of anti-VEGF-A DARPin. DARPin #4 (or PBS as control) was injected intravitreally into one eye of pigmented rabbits, while the other eye was left untreated. After 4 days, the treated eye was additionally administered human VEGF-A, while, again, the other eye was left untreated. If not inhibited, human VEGF-A induces vascular leakage in the rabbit eye. Two days after VEGF-A injection, fluorescein was given i.v. and 1 h post injection, the fluorescein leakage was recorded in both eyes. Comparing the treated with the untreated eye, the degree of VEGF-A inhibition can be derived. Upon complete VEGF-A inhibition, one would expect no vascular leakage in either the treated or the untreated eye, i.e. the fluorescence intensity ratio between treated and untreated eye is approximately 1. If no inhibition is taking place, a ratio of ≫1 is observed.

**Fig. 4:**
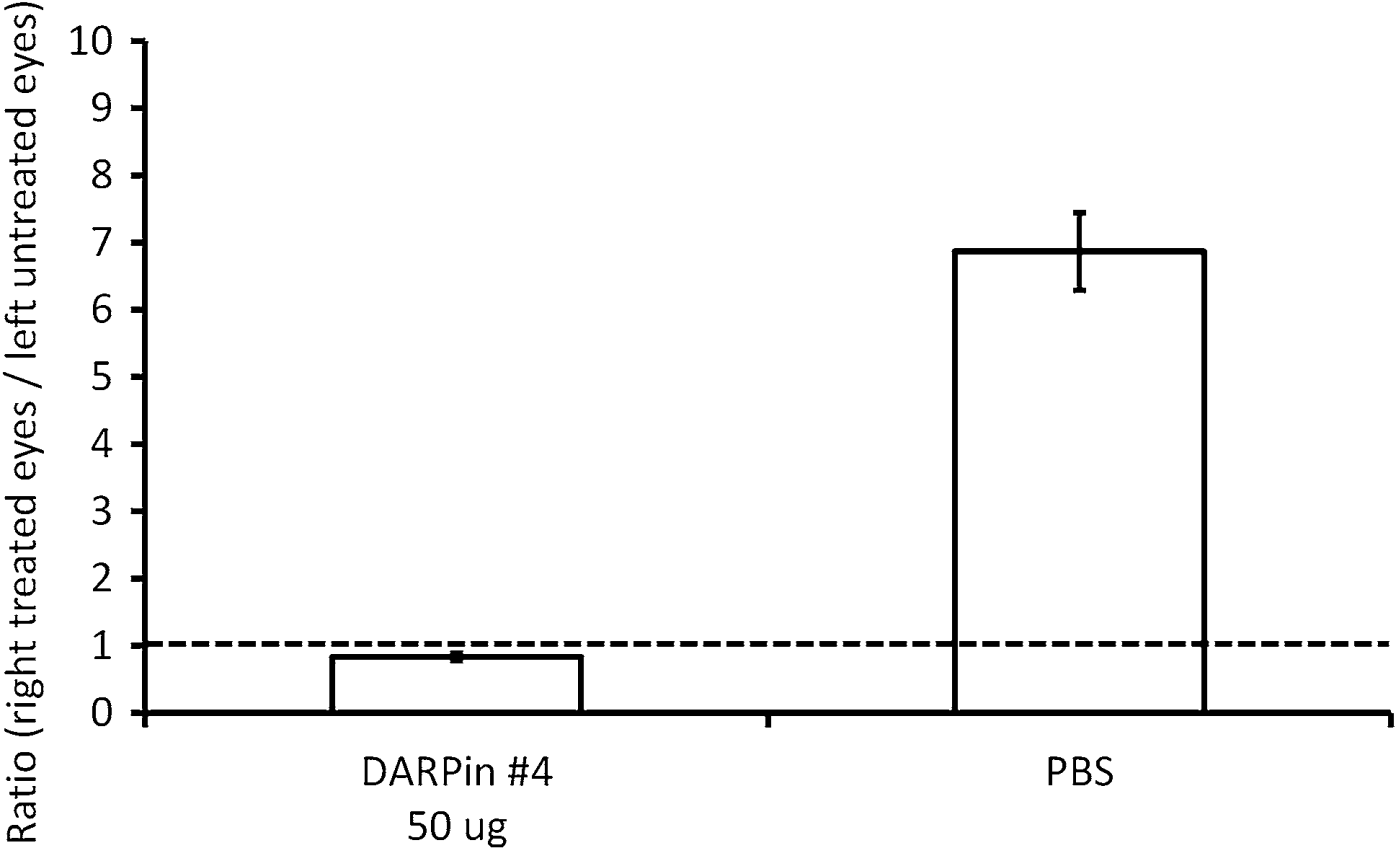
Inhibition of VEGF-A induced vascular leakage in rabbit eyes by intravitreal DARPin injection. The ratios of vascular leakage (fluorescein leakage in the eye) of the *right* (study) eyes in comparison to the *left* (untreated) eyes are shown. 50 μg DARPin #4 or PBS (in 100 μl volume each) were injected. The *dotted line* represents a ratio of ‘treated versus untreated eye’ of 1, which would be expected at complete inhibition of VEGF-A induced vascular leakage. Representative fluorescence scans are shown in Supplementary Figure 4

As shown in Fig. 4, intravitreal injection of 50 μg anti-VEGF-A DARPin significantly inhibits VEGF-A induced vascular leakage compared to control (*p* = 0.025), with a fluorescence leakage ratio of 0.8 ± 0.1. The vehicle control group showed a ratio of 6.9 ± 3.1. These results demonstrate almost complete inhibition of vascular leakage in DARPin-injected eyes and thus provide evidence for a potent inhibition of retinal VEGF-A effects by intravitreally injected DARPin. It is noteworthy that the dose used here is significantly below the clinically used dose of ranibizumab (500 μg/50 μl in human).

For studies using intravitreal injections, the potential for severe local side effects is higher compared to topical application [35]. Intravitreal injection of biologics therefore requires good compound purity in order not to induce inflammatory responses to for example bacterial endotoxins or other impurities. Rabbit eyes in particular are known to be strongly sensitive to even low levels of impurities in case of intravitreal injections. We therefore looked carefully for signs of inflammatory changes following intravitreal injections (corneal haze or clouding, intraocular flare, cataract formation etc.). We did not observe any signs of ocular inflammation or tissue damage both during in vivo examinations during the experimental time frame or upon histology analysis after completion of our experiments. No systemic side effects were noted at any time.

### Angiogenesis inhibition by topical DARPin application

**Fig. 5:**
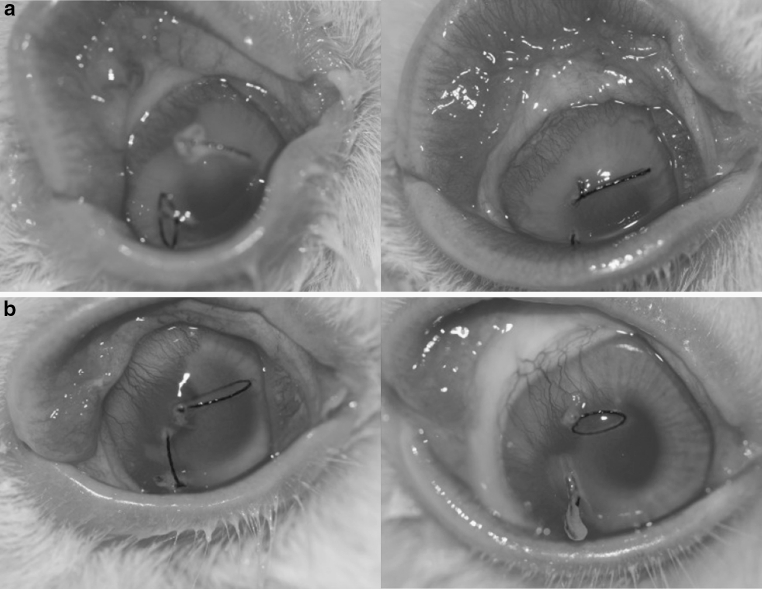
Prevention of blood vessel growth using DARPins in a rabbit corneal suture model. **a** Two eyes treated with DARPin #4 eight times daily in 50 μl drops at 20 mg/ml for 20 days. **b** Corresponding PBS-treated eyes

To assess the potential of using DARPins for topical anti-angiogenic treatment we used the well-established model of suture-induced corneal angiogenesis [36, 37]. Corneal angiogenesis was induced in rabbit eyes by placing sutures in the corneal stroma. Topical treatment consisted of anti-VEGF-A DARPin eye-drops or sham eye-drops for 20 days. The angiogenic response to the chronic suture stimulus was assessed by quantifying the length and thickness of blood vessels infiltrating the normally avascular cornea at the end of treatment. While vehicle-treated eyes showed substantial infiltration of corneal tissue by blood vessels, DARPin–treated eyes exhibited both a reduced length and density of infiltrating vessels (Fig. 5). Vessel density was reduced by 43 % and the maximum length of vessels was reduced by 31 %. These results demonstrate that topical application of anti-VEGF-A DARPin effectively attenuates corneal neovascularization.

**Fig. 6:**
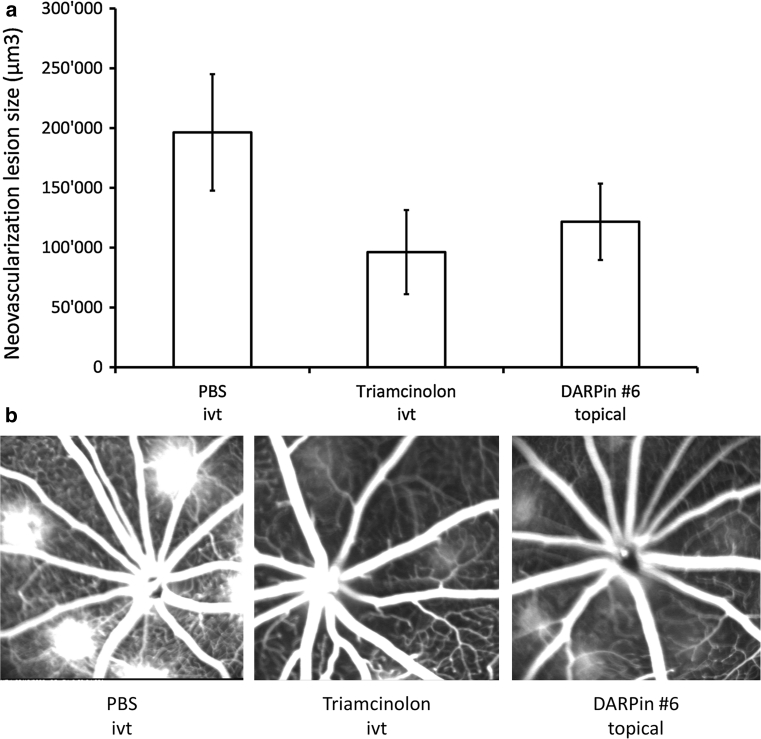
The effect of DARPin eye-drops on rat choroidal neovascularization (CNV) in comparison to Triamcinolon (positive control, ivt) [45] or PBS (buffer control, ivt). **a** Comparison of mean CNV lesion size of the different study groups. Neovascularization in eyes treated with Triamcinolon and topical DARPins was significantly reduced compared to PBS 24 days after Laser injury (*p* = 0.0065 for Triamcinolon and *p* = 0.025 for DARPin #6). **b** Representative pictures of CNV formation at day 15 taken with a Heidelberg retinal angiograph

To go one step further, we examined the effect of topical application of an anti-VEGF DARPin on the formation of new blood vessels in a rat model of laser-induced choroidal neovascularization (CNV). Topically applied DARPin #6 inhibited laser-induced CNV formation to a similar extent as Triamcinolone, which has previously been found to inhibit CNV formation in this model [38] (Fig. 6). Comparison of mean CNV lesion size of the different study groups shows significantly reduced neovascularization in eyes treated with Triamcinolon (*p* = 0.0065) and topical DARPin (*p* = 0.025) on day 15 after laser injury compared to PBS. This indicates the potential of topically applied anti-VEGF DARPin to treat chorioretinal neovascularization.

In both studies, the safety and tolerability of DARPins were assessed. Rabbits receiving topical DARPin gained weight normally, similar to PBS-treated rabbits (+25 % within experimental period). As the topical model included corneal sutures and associated corneal inflammation, intraocular ophthalmic findings are difficult to assess in vivo. Side effects included conjunctival chemosis, conjunctival discharge and conjunctival redness as well as corneal opacity at the sites of the sutures. As DARPin and PBS showed equal numbers of these minor conjunctival irritations, the findings are most likely procedure-related rather than compound-related. Note that no antibiotics were used in the study. No findings were observed in rat over the entire experimental period indicating DARPins were safe and well tolerated.

### The next generation of ophthalmic biologics

The next generation of VEGF inhibitors for ocular applications must offer significant advantages over the currently licensed products. Ideally, the administration should be simplified and the frequency of intraocular injections should be reduced. As a first step toward such drugs, we have generated anti-VEGF-A DARPins exhibiting single-digit picomolar potency. We showed that these anti-VEGF-A DARPins can specifically and potently inhibit angiogenesis and vascular leakage in vitro and in vivo. Compared to currently approved anti-VEGF compounds, DARPins display a significantly increased potency in functional models of angiogenesis. Notably, both VEGF-induced retinal hyperpermeablity as well as suture-induced corneal angiogenesis are efficiently suppressed by anti-VEGF-A DARPins administered either intravitreally or topically as eye-drops. The robust nature of DARPins allowed for simple liquid formulation and DARPins were well tolerated in different species in animal experiments. These DARPins thus represent attractive starting points for the development of novel anti-VEGF drugs as vascularization and vascular leakage inhibitors.

Topical application, as demonstrated in the present article and shown by others [15, 16], has the potential to be an attractive alternative to intravitreal injection for treatment of VEGF-driven retinal diseases. Intravitreal injection may have injection-related side effects, and avoiding this risk would be a great benefit for the patient. In addition, intravitreal injections have to be performed by health-care professionals and the associated costs with repetitive injections are substantial [13, 14]. Topical treatment of posterior segment disease with eye drops has the potential to both cut these costs and improve the safety of the procedure. The high stability and potency of DARPins presented in this study could represent a critical advantage in this respect. On top, topical DARPin could also be interesting for the treatment of corneal and anterior chamber diseases involving VEGF-A.

In summary, this study shows that a large set of highly potent VEGF-A inhibiting DARPins can rapidly be generated, and different DARPins show high efficacy in various in vitro and in vivo experiments. The results of the present study inspired the research and development of MP0112/AGN-150998, a VEGF-inhibiting DARPin which is currently being investigated in clinical trials for the treatment of wet age-related macular degeneration and diabetic macular edema, where data on both safety and efficacy, respectively, will be obtained.

## Materials and methods

### Ribosome display and binder screening, production and purification

Ribosome-display selections were performed using the N2C and N3C DARPin libraries essentially as described previously. In brief, two to three standard selection rounds were performed on VEGF-A immobilized in Maxisorp (Nunc) plates. The enriched DARPin library pools were subjected to three consecutive off-rate selection rounds using decreasing concentrations of biotinylated VEGF-A (1 nM to 10 pM) in combination with up to 1 μM free VEGF for competition. Binders were finally enriched using one additional standard selection round.

The resulting pools of DARPins were PCR amplified and ligated in pMPAG6 [39]. *E. coli* XL1-Blue was transformed with the resulting plasmid pool. Individual colonies were picked and grown overnight in 96-deep-well plates at 37 °C, and the resulting colonies were used to inoculate expression cultures in deep-well plates, and to isolate the plasmid DNA, as has been described [23]. Expression cultures were harvested by centrifugation, lysed, the crude extracts were applied to VEGF-A coated Maxisorp plates, and binding DARPins were detected using an anti-RGSHis antibody (QIAgen)/Anti-mouse IgG-AP conjugate (Sigma) detection system in combination with a 4-NPP (Sigma) detection system as described [23]. The DNA sequences of DARPins giving a crude extract ELISA signal were determined by standard DNA sequencing. The amino acid sequences of the DARPins are shown in Supplementary Figure 3.

Once the suitable DARPins were identified, DARPins were expressed and purified at larger scale as described [17, 40]. The purified proteins were re-buffered to PBS by dialysis (Mw cut off 3 kDa). Protein concentration was determined using the theoretical extinction coefficient of the respective DARPin at 280 nm. The purified DARPins were further characterized by SDS 15 %-PAGE, size exclusion chromatography (Superdex 200, GE Healthcare, Switzerland), and mass spectrometry. Note that all DARPins were bare single-domain DARPins, i.e. no fusion protein or chemical modification was used.

### Interaction analyses

Purified DARPins were analyzed by different ELISAs. For the ELISA analysis, purified DARPins at 10 nM final concentration were incubated with either PBS or 100 nM final concentration of human VEGF-A165, VEGF-A121 or VEGF-C [41] for 2 h at 4 °C. The mixtures were then applied for 20 min to Maxisorp plates coated with VEGF-A, followed by washing of the plate to remove VEGF and DARPin/VEGF complexes. Binding DARPins were detected using an anti-RGS-His antibody/anti-mouse-IgG-AP conjugate system as described [17].

DARPin VEGF-A-binding potencies were further assessed using a Quantikine kit (RnD Systems, UK) (Fig. 1). In brief, VEGF-A is incubated with test substances, and the mixture is applied to an ELISA plate coated with an anti-VEGF-A monoclonal antibody. The amount of free VEGF-A, which binds to the plate, is subsequently detected using a polyclonal anti-VEGF-A antibody-HRP conjugate. For screening different inhibitor candidates VEGF-A at 25 pM was incubated with various test substances. All inhibitors and the isotype controls were applied at 250 pM, bevacizumab was applied at 125 pM as it is bivalent. Further controls contained 25 pM, 12.5 pM, 6.25 pM, and 0 pM VEGF-A only, respectively, without any inhibitor. For IC50 analysis of DARPin #4, 20 pM VEGF was used and values were measured in triplicates at room temperature. Data were fitted using Graphpad Prism (Graphpad, USA).

### Spheroid sprouting assay

Human umbilical vein endothelial cells (HUVECs, Promocell, Heidelberg, Germany) were used from passage 2–7 for sprouting experiments. Cells were cultured as monolayers at 37 °C, 5 % CO_2_ in a humidified atmosphere in Endothelial Cell Growth Media (ECGM, Promocell). The preparation of EC spheroids was performed as described previously [27–29, 42]. Briefly, cells were harvested from sub-confluent monolayers by trypsinization and suspended in ECGM containing 10 % FBS and 0.25 % (w/v) carboxymethylcellulose (Sigma). 500 cells were seeded together in one hanging drop to assemble into a single spheroid within 24 h at 37 °C, 5 % CO_2_. After 24 h spheroids were harvested and used for sprouting analysis in a matrix of type I collagen as previously described [27–29, 42]. Briefly, 30 EC spheroids per group were seeded into 0.5 ml collagen solution in non-adherent 24-well plates, with a final concentration of rat type I collagen of 1.5 mg/ml. Freshly prepared gels were transferred rapidly into a humidified incubator (37 °C, 5 % CO_2_) and after the gels had solidified, 0.1 ml serum-free Endothelial Cell Basal Media (ECBM, Promocell) was added per well containing VEGF (3 ng/ml; about 160 pM VEGF-A monomer), and DARPins or controls (ranibizumab, isotype control DARPins) at 1 nM, or PBS, respectively. After 24 h, gels were photographed and spheroid sprouting was assessed quantitatively. 10 spheroids were analyzed per condition. Results were calculated as mean ± SEM.

### Receptor phosphorylation assay

HUE (immortalized HUVEC) cells [43] were plated in ECGM supplemented with 10 % FCS with 35,000 cells/well in 48 well cell culture dishes. After 24 h the medium was exchanged against ECBM supplemented with 10 % FCS and kept overnight. Cells were then incubated with the 1.4 nM VEGF-A, a mixture of 1.4 nM test substance with 1.4 nM VEGF-A, or buffer control for 7 min at room temperature, immediately followed by cell lysis under conditions inhibiting phosphatase activity. Quantification of RTK-Phosphorylation was assessed in 96well plates via sandwich ELISA (OD_450–540 nm_) using a respective VEGF-R2 specific capture antibody and an anti-phosphotyrosine detection antibody [44].

### Intravitreal DARPin: mouse ocular penetration

DARPin #4 and isotype control DARPin I1, both with one C-terminal cysteine each, were expressed and purified as described above. The purified DARPins were re-buffered to PBS and labeled on the free cysteine with Alexa-555 (Sigma, Switzerland), according to the manufacturer’s protocol. The labeled DARPin was then separated from free label using a Nap-5 column followed by extensive dialysis against PBS. UV spectroscopy measurements indicated 32 and 73 % labeling efficiency each. Endotoxins were then removed using EndoTrap (Hyglos, Germany) columns. The labeled proteins were finally concentrated at 34 uM and 130 uM, respectively.

For intravitreal injection, 3 week old mice were anesthetized with isoflurane and after pupil dilation, DARPins or vehicle (PBS) control (1 μl) were injected under an operating microscope into the vitreous cavity. Mice were sacrificed and eyes taken at the indicated time points (Supplementary Figure 2). The animal experiments adhered to the Association for Research in Vision and Ophthalmology (ARVO) Statement for the Use of Animals in Ophthalmic and Vision Research and were approved by the Animal Care and Use Committee at the University of Freiburg, Germany.

### Intravitreal DARPin: rabbit vascular leakage model

Two groups of 5 pigmented rabbits aged approximately 4 months (2–2.5 kg) were used. On day 1, animals were anesthetized with an intramuscular injection of xylazine (7.5 mg/kg) and ketamine (32 mg/kg). After cleaning each eye with betadine, a 100 μl single intravitreal injection of DARPin #4 (0.05 mg) or vehicle into the right eyes was placed about 3 mm posterior to the limbus in the supratemporal quadrant of the eye using a 30G needle. The intravitreal injection was performed under an operating microscope on dilated eyes (1 drop of 10 % phenylephrine and 1 drop of 0.5 % tropicamide) instilled 15–20 min before the injection. On day 4, with an equivalent procedure, animals received a single intravitreal injection of 50 μl (500 ng) recombinant human VEGF-A165 into the right eyes. On day 6, 47 h after VEGF-A challenge, 10 % sodium fluorescein (50 mg/kg in 0.9 % saline) was injected via the marginal ear vein of conscious animals. One hour later, the fluorescein content in the vitreoretinal compartment was assessed using an FM-2 Fluorotron Master ocular photometer on both eyes. Rabbits were anesthetized with an intramuscular injection of 32 mg/kg ketamine and 7.5 mg/kg xylazine and pupils were dilated with one drop of 10 % phenylephrine and one drop of 0.5 % tropicamide 20 min prior to the examination. A series of scans of 148 steps (step size 0.25 mm) were performed from the cornea to the retina along the optical axis. The ratios of AUCs between the right treated and the left untreated eyes were calculated for each animal. The assessment of ocular tolerability was performed on days 2 and 4 using a slit lamp and vitreal clarity was assessed using a Nussenblatt’s score. Body weight was measured before the start of the study on day 1 and prior to termination of the animals on day 6. Animal behavior was assessed daily.

### Topical DARPins: rabbit corneal suture

New Zealand white rabbits were anesthetized with an intramuscular injection of ketamine (32 mg/kg) and xylazine (7.5 mg/kg) and a lid speculum was inserted to facilitate the procedure. Two 3 mm long 7–0 silk sutures were placed in temporal and superior area of the cornea on day 1 (4 mm from the limbus at 9 and 12 o’clock). The eyes (four each) were treated with DARPin in PBS or PBS only eight times daily (8 h doses during daytime, 17 h interval at nights) from Days 1 to 20. The DARPin stock solution was concentrated to 20 mg/ml, and 50 μl were applied per instillation (1 mg/instillation). The extent of corneal neovascularization was evaluated by the maximum vessel length (ratio vessel length/distance from limbus to suture) and the density of new vessels in the neo-vascularized area. Images of every eye were recorded twice a week, and representative pictures prior to termination are shown in Fig. 5. Eyes were evaluated using an ophthalmoscope before treatment, just following the first and the last administration on days 1–3 and twice a week up to Day 20, and were evaluated using a Draize score. Body weight was recorded before induction and treatment (baseline), on the day of induction (day 1) and then once a week until day 20.

### Topical DARPins: rat choroidal neovascularization

Brown Norway rats were divided into 6 groups of 6 animals each. Choroidal neovascularization was induced using a 532 nm argon laser for photocoagulation (six 75 μm-sized spots at 150 mW for 100 ms) in the right eyes on day 1. Topical DARPin #6 was administered by instillations (10 μl of 10 mg/ml) 4 times a day (8:00 am, 12:00 am, 4:00 pm, 8:00 pm) from day 1. Vehicle (PBS) or reference (Triamcinolon; 120 μg) were administered by a single intravitreal injection (3 μl; post induction on the day of induction, i.e. day 1). Angiography was performed on days 15 and 22 post induction, 10 min after subcutaneous fluorescein injection.

Ocular observations were made on both eyes of all animals using an ophthalmoscope and an evaluation was performed using a 3-level scale (0, 1, 2). Both eyes were examined on Day 1 just after treatment and just after the last administration, on Day 2 before the first and after the last instillation (the ivt groups were examined at the same time), and then after the last administration on Days 8, 15 and 22. After sacrifice on Day 24, the treated right eye from all animals was sampled, the choroid was flatmounted and the volume of the CNV was evaluated and quantified microscopically using a Zeiss ApoTome and z-stack reconstruction with the Axiovision 4.6 software (Carl Zeiss).

## Electronic supplementary material

Below is the link to the electronic supplementary material.

Supplementary material 1 (DOC 10617 kb)
