# Supplementary material for: Highly potent VEGF-A-antagonistic DARPins as anti-angiogenic agents for topical and intravitreal applications
Source: Angiogenesis. 2012 Sep 15;16(1):101–11. doi: 10.1007/s10456-012-9302-0 (PMC3526737; doi:10.1007/s10456-012-9302-0)
Supplement: Supplementary file 1 — Supplementary material 1 (DOC 10617 kb) [file 10456_2012_9302_MOESM1_ESM.doc]

# Supplementary Figures

# Highly potent VEGF-A-antagonistic DARPins as anti-angiogenic agents for topical and intravitreal applications

Andreas Stahl^1*^, Michael T. Stumpp^2*^, Anja Schlegel^2^, Savira Ekawardhani^2^, Christina Lehrling^1^, Gottfried Martin^1^, Maya Gulotti-Georgieva^2^, Denis Villemagne^2^, Patrik Forrer^2^, Hansjürgen T. Agostini^1^, & H. Kaspar Binz^2†^

^1^ Universitäts-Augenklinik Freiburg, Killianstrasse 5, 79106 Freiburg, Germany

^2^ Molecular Partners AG, Wagistrasse 14, 8952 Zürich-Schlieren, Switzerland

**Running title:**

Highly potent VEGF-A antagonistic DARPins

**Keywords:**

angiogenesis, DARPin, ophthalmology, potency, VEGF

**Conflict of interest:**

MTS, PF, and HKB are shareholders of Molecular Partners AG, commercializing the DARPin technology; all other authors do not declare any conflict of interest other than their affiliations.

**Author contributions:**

All authors performed research. ASt, MTS, PF, HTA and HKB designed research and analyzed the data, ASt and HKB wrote the manuscript.

^†^ To whom correspondence should be addressed:

Dr. H. Kaspar Binz

Molecular Partners AG

Wagistrasse 14

8952 Zürich Schlieren

Tel: +41 44 755 77 00

Fax: +41 44 755 77 07

E-mail: kaspar.binz@molecularpartners.com

^*^ These authors contributed equally to this work

| 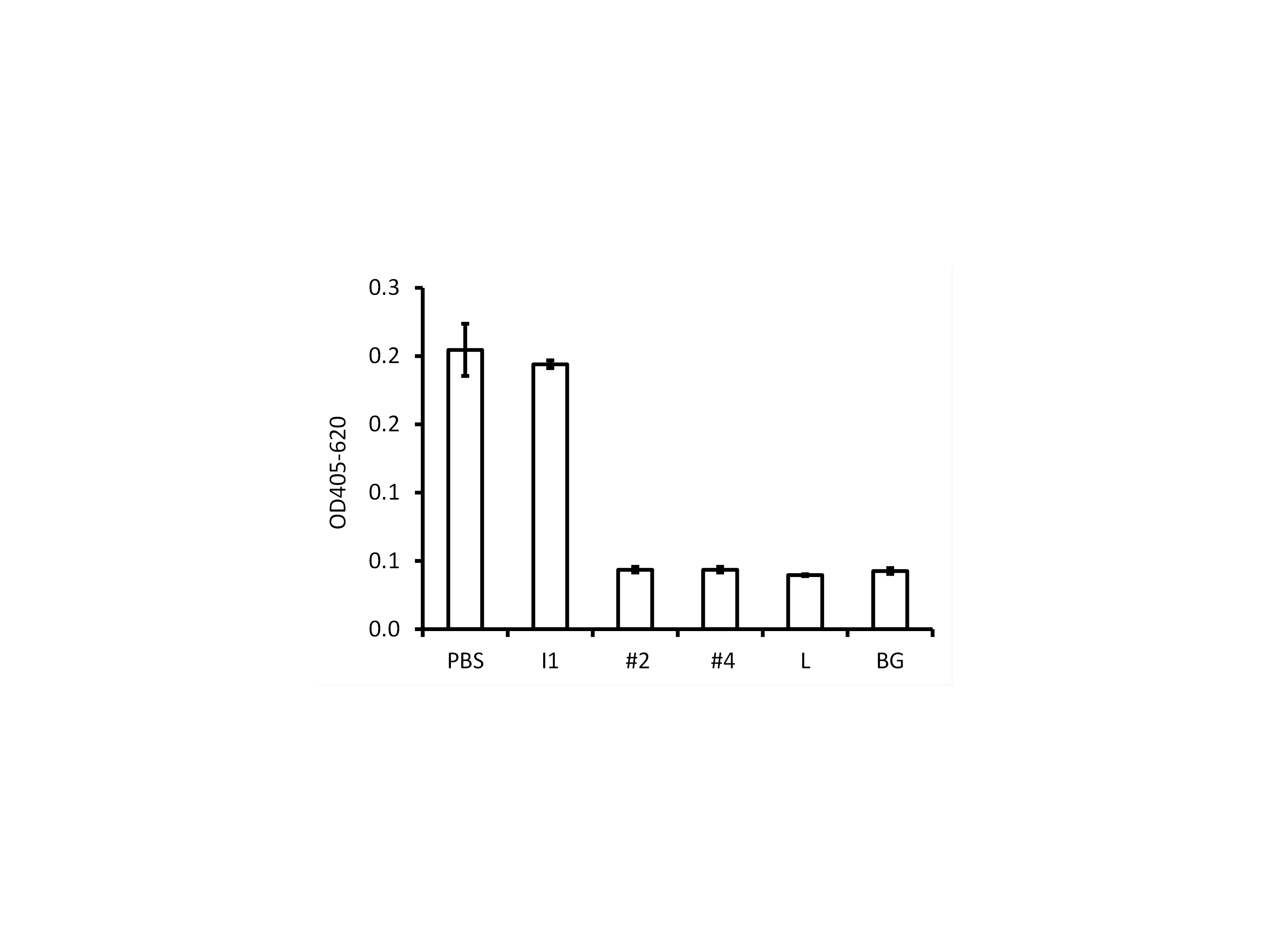 |
| --- |
| **Supplementary Figure 1.** Receptor interference ELISA. PBS, the DARPins I1, #2 or #4, as well as ranibizumab (L; all at 100 nM) were pre-incubated with 10 nM of biotinylated VEGF-A for 2 h at 4°C. The mixture was then applied to a Maxisorp (Nunc) plate coated with 60 nM human VEGFR-2 and blocked with 0.5% BSA in PBST for 20 minutes and binding VEGF-A was detected using a streptavidin-AP (Roche) conjugate. The column BG represents background signal of the ELISA plate from a well not coated with VEGFR-2. This experiments shows that DARPins #2 and #4 and ranibizumab interfere with the binding of VEGF-A to its receptor, reducing the signal to virtually background (BG). DARPin I1 does not interfere with the VEGF-A/VEGFR-2 interaction giving an equivalent result as PBS. |

| (a)  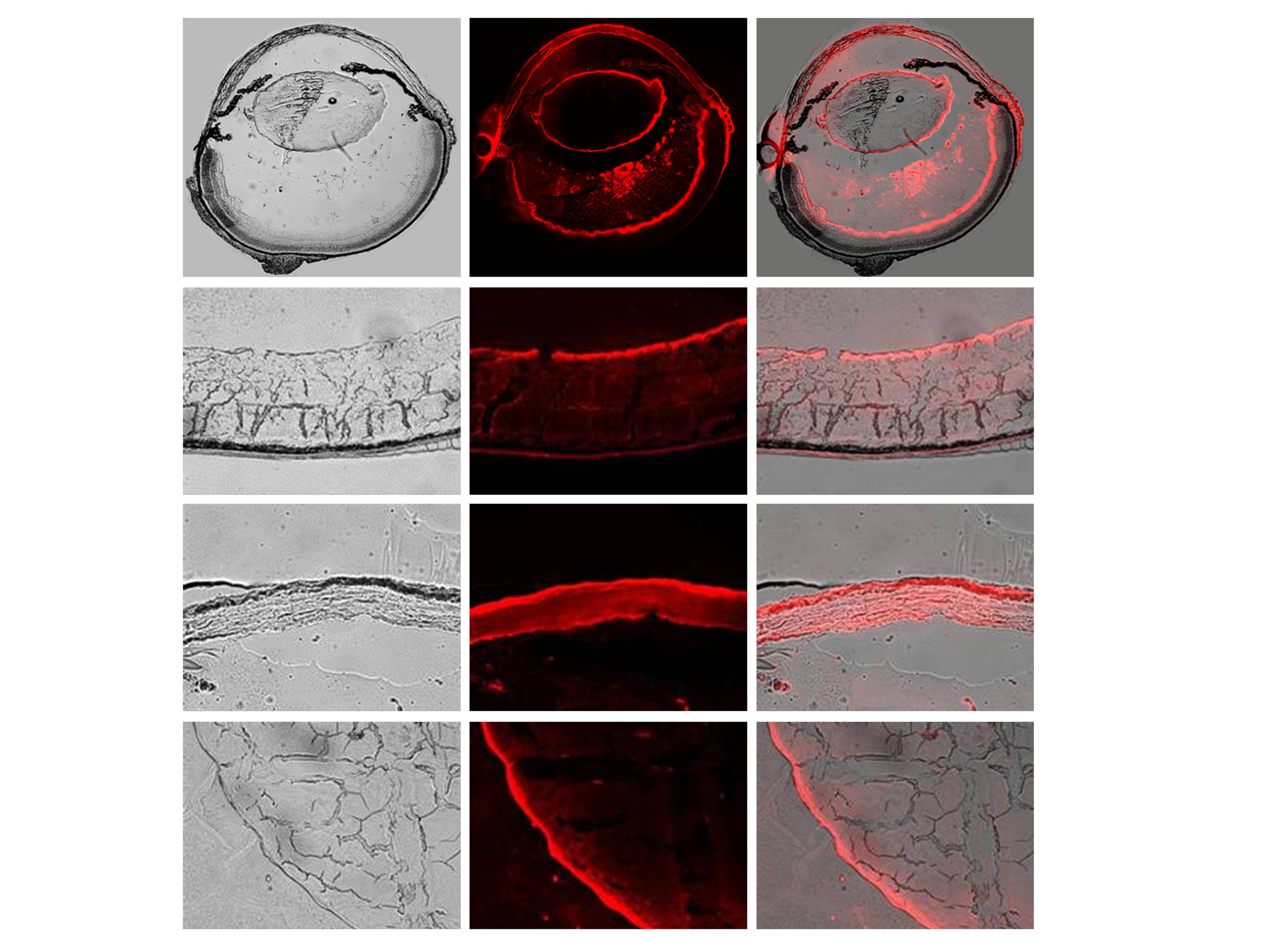  (b)  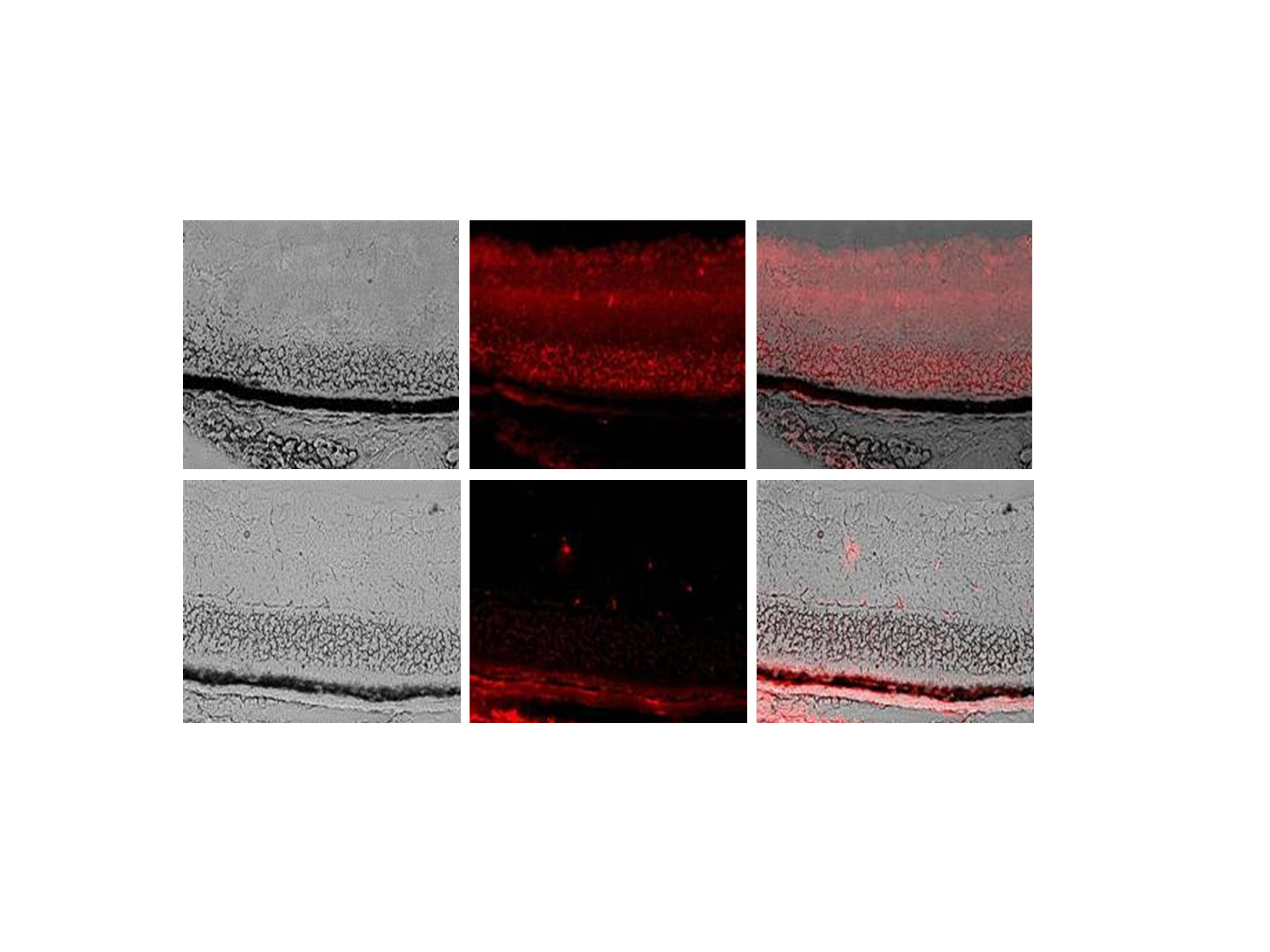 |
| --- |
| Supplementary Figure 2. Ocular tissue penetration of fluorescently labeled DARPins after intravitreal injection. Light microscope images (left column), fluorescent images (central column), and overlay images (right column) are shown. (a) Top row: 5x overview images of Alexa555-labeled DARPins 30 minutes after intravitreal injection into adult mouse eyes. Injection was intravitreally at the left side of the eye. Second to fourth row: 20x images of retina, cornea and lens from the same eye. (b) Top row: Penetration of fluorescent DARPins into all layers of the retina 24 h after intravitreal injection. Bottom row: Retina of vehicle-injected eyes. Experimental details are described in the Materials and Methods section. |

|  |
| --- |
| **Supplementary Figure 3.** Amino acid sequences of the DARPins used in the present study. |

| 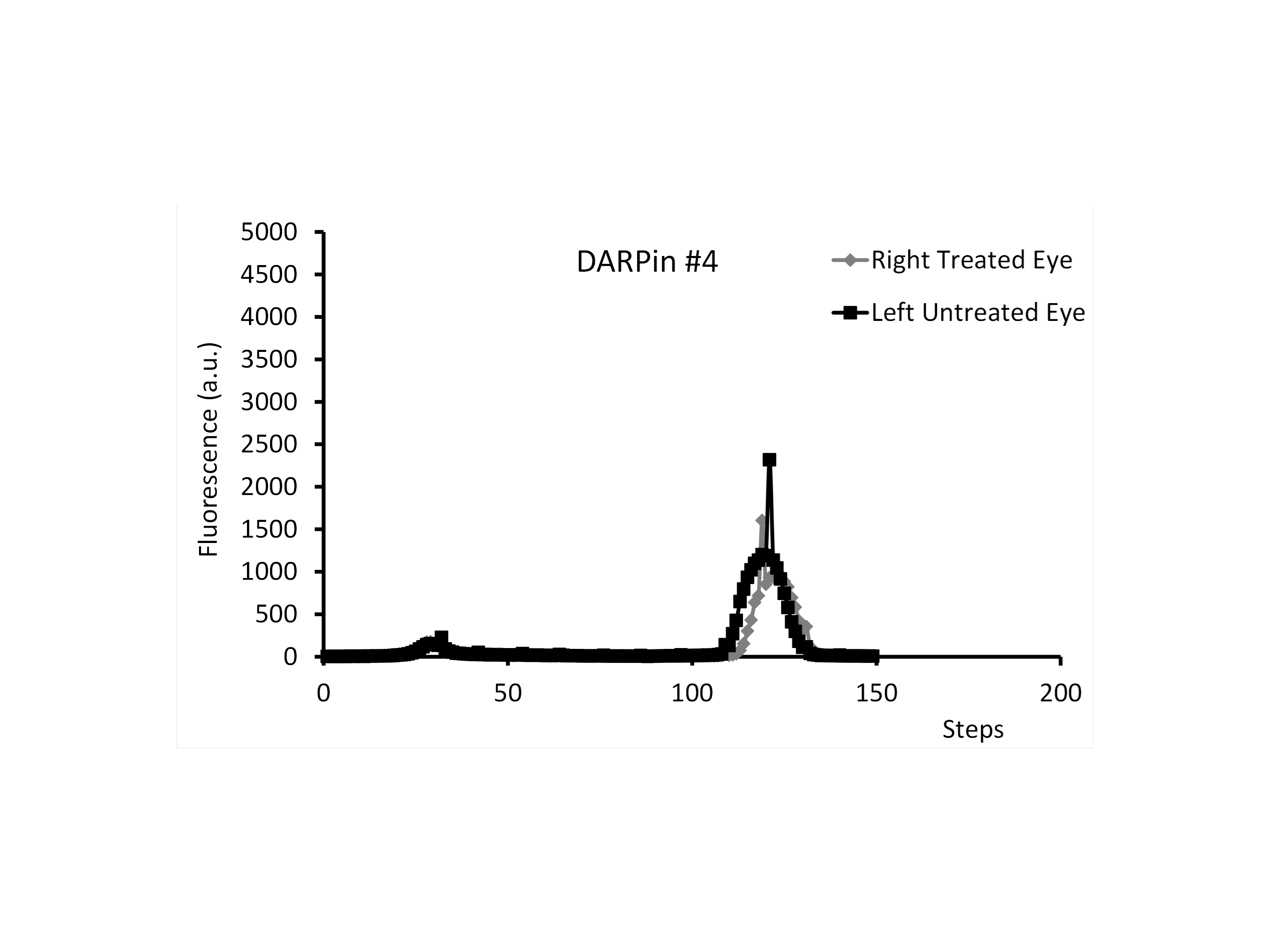  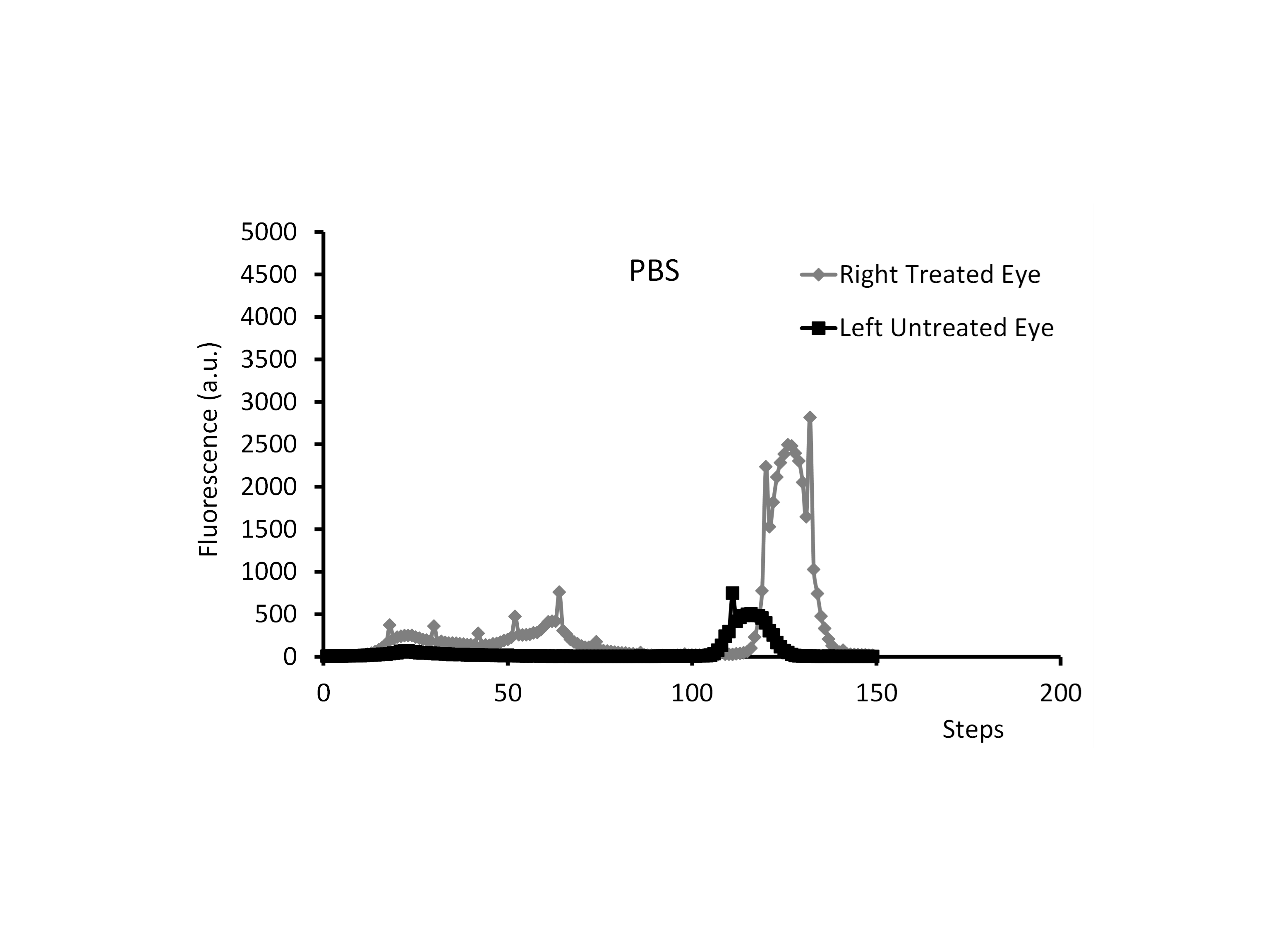 |
| --- |
| **Supplementary Figure 4.** Representative fluorescence scans of the rabbit vascular leakage experiment (see Figure 4). (a) DARPin #4 inhibits vascular leakage, resulting in equivalent amounts of leaking fluorescein in the right (VEGF-treated) and the left (untreated) rabbit eye. (b) In case of PBS, the right (VEGF-treated) eye shows significantly larger amounts of leaking fluorescein than the left (untreated) eye. |
